# Anti-obesity therapy for cardiovascular disease prevention: potential expected roles of glucagon-like peptide-1 receptor agonists

**DOI:** 10.1186/s12933-022-01611-8

**Published:** 2022-09-06

**Authors:** Kosuke Sawami, Atsushi Tanaka, Koichi Node

**Affiliations:** 1 Department of Cardiovascular Medicine, Graduated School of Medicine, The University of Tokyo, Tokyo, Japan; 2 Department of Cardiovascular Medicine, Saga University, 5-1-1 Nabeshima, Saga, 849-8501 Japan

**Keywords:** Obesity-related cardiovascular disease, Semaglutide, Liraglutide, Glucagon-like peptide-1 receptor agonists, Anti-obesity

## Abstract

Obesity is characterized by visceral fat accumulation and various metabolic disturbances that cause metabolic syndrome and obesity-related cardiovascular diseases (ORCVDs). Hence, treatments targeting obesity should also prevent ORCVDs. Nonetheless, lifestyle modification therapy alone is still insufficient to reduce the risk of ORCVDs, although most cardiovascular guidelines still list it as the only treatment for obesity. Additionally, conventional anti-obesity drugs, such as orlistat, phentermine-topiramate, and naltrexone-bupropion, can reduce body weight but have not demonstrated a clear reduction in the risk of ORCVDs. To overcome this unmet clinical need, newer anti-obesity drugs must exhibit not only sufficient and long-lasting weight loss but also obvious cardiovascular benefits. Given recent clinical findings and evidences, in this context glucagon-like peptide-1 receptor agonist is currently available as a candidate that is clinically positioned as a first-line anti-obesity agent for the effective prevention of ORCVDs in people with obesity.

Obesity is characterized by visceral fat accumulation and various metabolic disturbances that cause metabolic syndrome and obesity-related cardiovascular diseases (ORCVDs). Excess adipose tissue in visceral fat releases proinflammatory adipokines and suppresses the secretion of anti-inflammatory adipocytokines. Subsequently, low-grade systemic and chronic inflammation occurs, which leads to insulin resistance and vascular failure, both of which are essential in the pathogenesis of atherosclerosis [1]. Hence, treatments targeting obesity should also prevent ORCVDs. However, treatment interventions for obesity are almost exclusively limited to lifestyle modification. Even in the United States, where several anti-obesity drugs have been approved, the prescription rates for these drugs are very low, and most cardiovascular guidelines still recommend lifestyle modifications as the only treatment for obesity to prevent ORCVDs [2–4].

Lifestyle modification through low-fat and low-calorie diets and increased physical activity generally result in a 3–10% loss of initial body weight [2]. More than 5% weight reduction improves insulin sensitivity in multiple tissues and pancreatics beta cell function, ameliorating glycemic and lipid profiles and decreasing blood pressure [2, 5]. However, there was no significant reduction in the risk of adverse cardiovascular events or mortality in a previous systematic review and meta-analysis of clinical trials examining the effects of lifestyle modification [6]. In the Look AHEAD study, which examined the effect of intensive lifestyle intervention in patients with obesity and type 2 diabetes, the composite endpoint of adverse cardiovascular events and mortality was reduced by approximately 20% over a median follow-up period of 10.2 years, only in the group of patients that achieved a weight loss of 10% or more from their initial body weight (about 40% of the intervention arm) [7, 8]. In addition, bariatric surgery has been suggested to reduce body weight by more than 20% and cardiovascular mortality by approximately 60% [1, 9]. These findings suggest that the degree of weight loss is associated with the preventive effect of these adverse events and that at least 10% weight loss is likely necessary to prevent them.

Anti-obesity medications such as orlistat, phentermine-topiramate, and naltrexone-bupropion have induced a weight loss of no more than 10%, which is insufficient to prevent the risk of ORCVDs. None of these drugs have demonstrated a clear effect in improving cardiovascular outcomes and reducing mortality risk, partly because they only have an anorexic effect [2]. To compensate for its moderate weight loss effect, anti-obesity drugs used to prevent ORCVDs are also expected to benefit the cardiovascular system through non-anorexic mechanisms. Additionally, the duration of the weight loss effect is an important issue. With lifestyle modification alone, the weight loss effect is limited to approximately one year, after which weight increases due to endocrine mechanisms involved in body weight regulation [6, 10]. Taken together, the possible requisites of anti-obesity drugs for ORCVDs prevention are the degree of weight loss, the effect of maintaining body weight, and the unique effect of cardiovascular protection (Fig. 1).

**Fig. 1:**
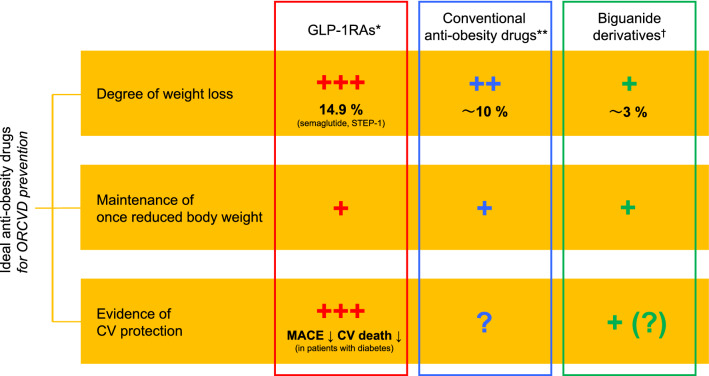
Requisites of anti-obesity drugs for ORCVD prevention. Of the three ideal conditions for anti-obesity drugs to prevent ORCVDs, GLP-1RAs appear to have advantages over conventional drugs and biguanide derivatives in terms of the degree of weight loss (especially semaglutide) and specific cardiovascular effects. *Mainly semaglutide and liraglutide. **Orlistat, phentermine-topiramate, and naltrexone-bupropion. ^†^Mainly metformin. CV, cardiovascular; GLP-1RAs, glucagon-like peptide-1 receptor agonists; MACE, major adverse cardiovascular events; ORCVD, obesity-related cardiovascular disease; STEP, Semaglutide Treatment Effect in People with Obesity

Glucagon-like peptide-1 receptor agonists (GLP-1RAs), developed as glucose-lowering drugs, beneficially modify several cardiovascular risk factors, such as blood pressure, HbA1c, and body weight, and reduce the risk of major adverse cardiovascular events (MACE) [11]. GLP-1RAs may be potential anti-obesity drugs that satisfy the requirements of anti-obesity drugs for ORCVD prevention. Thus, GLP-1RAs are potential anti-obesity drugs that satisfy the requirements for ORCVD prevention. Recent large-scale clinical trials have been conducted to verify the weight loss effects of GLP-1RAs, especially liraglutide and semaglutide, in patients without diabetes [12, 13], which showed statistically significant weight loss when compared with lifestyle modification only. In the Satiety and Clinical Adiposity − Liraglutide Evidence (SCALE) trial [12], patients allocated to receive 3 mg liraglutide lost 8.0% of their body weight from baseline, whereas those in the placebo group lost 2.6%. The percentage of patients who lost at least 5% or 10% of their body weight from baseline was 63.2% vs. 27.1% and 33.1% vs. 10.6% in the liraglutide and placebo groups, respectively [12]. In the Semaglutide Treatment Effect in People with Obesity (STEP)-1 trial [13], there was a statistically significant weight loss in the 2.4 mg semaglutide group compared to the placebo group, with a 14.9% reduction in body weight from baseline in the semaglutide group and 2.4% reduction in the placebo group. The percentage of patients who lost at least 5% vs. 10% of their body weight from baseline was 86.4% vs. 31.5% and 69.1% vs. 12.0% in the semaglutide and placebo groups, respectively [13]. Notably, the degree of weight loss caused by semaglutide exceeds 10% [13]. These results suggest that semaglutide has the potential to reduce the risk of ORCVDs, although its effect on weight loss falls short of that of bariatric surgery. Currently, the Semaglutide Effects on Cardiovascular Outcomes in People with Overweight or Obesity (SELECT) study that assesses the effect of semaglutide on cardiovascular events in non-diabetic patients with obesity and established cardiovascular diseases is ongoing [14]. Recently, 15 mg tirzepatide, a novel dual glucose-dependent insulinotropic polypeptide (GIP) and glucagon-like peptide-1 (GLP-1) receptor agonist, reduced the body weight of patients with obesity by 20.9% from baseline compared with placebo in the SURMOUNT-1 trial [15]. Since this weight loss effect was comparable to that of bariatric surgery, tirzepatide therapy is also expected to reduce the risk of ORCVDs.

GLP-1RAs are thought to exert their weight loss effects by suppressing feeding rather than increasing energy expenditure. GLP-1 receptors are expressed in proopiomelanocortin/cocaine- and amphetamine-regulated transcript neurons in the arcuate nucleus of the hypothalamus and are considered to play a role in GLP-1-mediated regulation of feeding. When liraglutide was administered peripherally, it was observed to bind to the arcuate nucleus in rats. Functional magnetic resonance imaging in humans demonstrated that peripheral administration of GLP-1 suppresses activity in the hypothalamus, amygdala, insula, and orbitofrontal cortex, thereby decreasing appetite [16]. However, the concentration of L-cell-derived endogenous GLP-1 in the blood is quite low. L cell-derived endogenous GLP-1 has been suggested to act locally on ileal enteric neurons rather than as a circulating hormone, causing stomach distention and inhibiting feeding by stimulating hypothalamic neurons via the spinal afferent pathway [17]. Endogenous GLP-1 is also secreted by GLP-1-producing neurons in the nucleus tractus solitarii (NTS), so there may be several pathways to regulate appetite via endogenous GLP-1 and GLP-1RAs [16].

Regardless of the patient's perseverance, endocrinological factors make it challenging to maintain weight once it is lost. Weight loss induces a decrease in energy expenditure and changes in appetite perception, which leads to weight gain. Since endocrinological changes such as changes to leptin, ghrelin, and GIP levels that promote weight regain persist long after weight loss [10], patients with obesity regain weight after one year of lifestyle modification. Therefore, lifestyle modification, which is mainly “education” of patients, cannot prevent weight regain, and intervention with drugs that can modify endocrinological changes is necessary. The SCALE Maintenance trial of liraglutide and STEP-4 trial of semaglutide examined the long-term effects of GLP1-RAs on weight loss, both of which showed that the weight loss effect was sustained for up to 68 weeks with the continuation of these drugs, and discontinuation of these drugs resulted in weight gain [18, 19]. These results suggest that continuing GLP-1RAs has an inhibitory effect on weight regain and that they must be used long-term in treating obesity.

The major difference between GLP-1RAs and existing anti-obesity drugs is that GLP-1RAs are assumed to have favorable cardiovascular effects, although whether GLP-1RAs act directly or indirectly on the cardiovascular system is currently unknown [20]. In previous cardiovascular outcome trials of liraglutide (LEADER) and semaglutide (SUSTAIN-6) in patients with diabetes who were at high risk for cardiovascular diseases, the risk of MACE was significantly reduced by 13% and 26%, respectively, when compared with placebo [21]. A meta-analysis including other GLP-1RAs also showed a 14% reduction in MACE and a 13% reduced risk of cardiovascular mortality when compared to placebo in patients with diabetes [22]. The expression of the GLP-1 receptor in vascular smooth muscle cells and endothelial cells remains unclear. Nevertheless, in vitro studies have demonstrated the antiproliferative effects of GLP-1 and GLP-1RAs treatments on vascular smooth muscle cells, reductions in reactive oxygen species generation, and increases in nitric oxide formation in endothelial cells [20]. Collectively, these cardioprotective effects may be due to the direct action of GLP-1RAs and indirect action through anti-inflammation, weight reduction, or both.

Metformin, which has been widely used in the treatment of diabetes, has recently been reconsidered for its effects of weight loss and cardiovascular protection. In the Diabetes Prevention Program (DPP) and Diabetes Prevention Program Outcomes Study (DPPOS), metformin group showed 2.7% weight loss from baseline compared to placebo at 1 year, and this effect was maintained at 10 years [23]. Metformin is thought to have an anorexic effect partly by promoting the secretion of GLP-1 and activating afferent inputs from the intestinal tract to the NTS [24], but there is currently no evidence that the degree of weight loss exceeds 5%, which is the standard for use as an anti-obesity drug. Metformin reduced cardiovascular events in new-onset diabetes patients with obesity in the UK Prospective Diabetes Study (UKPDS 34) [25], but not in DPP/DPPOS [26], and some meta-analyses have shown controversial results [27, 28]. Thus, the cardiovascular protective effect of metformin is still inconclusive. The VA-IMPACT (NCT02915198) is ongoing to evaluate the effect of metformin on the prevention of cardiovascular events in patients with impaired glucose tolerance and established cardiovascular disease, but at the moment, the strength of evidence of metformin as a drug to prevent ORCVD may be inferior to GLP-1RAs.

As mentioned above, clinical trials, mainly for patients with type 2 diabetes, have confirmed that GLP-1RAs induce sufficient long-lasting weight loss effects and specific favorable effects on cardiovascular systems. These effects are likely prerequisites for obesity treatment to reduce the incidence of ORCVDs. The results of ongoing clinical trials will determine whether GLP-1RAs can reduce weight loss and ORCVDs in non-diabetic patients with obesity. Although there are still unsolved clinical issues, such as the cost-effectiveness of the long-term prescription, GLP-1RAs are far less invasive than bariatric surgery and should be actively considered for use in the growing number of patients with obesity.

## Data Availability

Not applicable.

## Abbreviations

DPP: Diabetes prevention program
DPPOS: Diabetes prevention program outcomes study
GIP: Glucose-dependent insulinotropic polypeptide
GLP-1RA: Glucagon-like peptide-1 receptor agonist
MACE: Major adverse cardiovascular events
NTS: Nucleus tractus solitary
ORCVD: Obesity-related cardiovascular disease
SCALE: Satiety and clinical adiposity − liraglutide evidence
STEP: Semaglutide treatment effect in people with obesity
